# Genetic, Ecological and Morphological Distinctness of the Blue Mussels *Mytilus trossulus* Gould and *M*. *edulis* L. in the White Sea

**DOI:** 10.1371/journal.pone.0152963

**Published:** 2016-04-04

**Authors:** Marina Katolikova, Vadim Khaitov, Risto Väinölä, Michael Gantsevich, Petr Strelkov

**Affiliations:** 1 Department of Ichthyology and Hydrobiology, Saint-Petersburg State University, Saint-Petersburg, Russia; 2 Department of Invertebrate Zoology, Saint-Petersburg State University, Saint-Petersburg, Russia; 3 Kandalaksha State Nature Reserve, Kandalaksha, Murmansk Region, Russia; 4 Finnish Museum of Natural History, University of Helsinki, Helsinki, Finland; 5 Department of Invertebrate Zoology, Lomonosov Moscow State University, Moscow, Russia; Australian Museum, AUSTRALIA

## Abstract

Two blue mussel lineages of Pliocene origin, *Mytilus edulis* (ME) and *M*. *trossulus* (MT), co-occur and hybridize in several regions on the shores of the North Atlantic. The two species were distinguished from each other by molecular methods in the 1980s, and a large amount of comparative data on them has been accumulated since that time. However, while ME and MT are now routinely distinguished by various genetic markers, they tend to be overlooked in ecological studies since morphological characters for taxonomic identification have been lacking, and no consistent habitat differences between lineages have been reported. Surveying a recently discovered area of ME and MT co-occurrence in the White Sea and employing a set of allozyme markers for identification, we address the issue whether ME and MT are true biological species with distinct ecological characteristics or just virtual genetic entities with no matching morphological and ecological identities. We find that: (1) in the White Sea, the occurrence of MT is largely concentrated in harbors, in line with observations from other subarctic regions of Europe; (2) mixed populations of ME and MT are always dominated by purebred individuals, animals classified as hybrids constituting only ca. 18%; (3) in terms of shell morphology, 80% of MT bear a distinct uninterrupted dark prismatic strip under the ligament while 97% of ME lack this character; (4) at sites of sympatry MT is more common on algal substrates while ME mostly lives directly on the bottom. This segregation by the substrate may contribute to maintaining reproductive isolation and decreasing competition between taxa. We conclude that while ME and MT are not fully reproductively isolated, they do represent clearly distinguishable biological, ecological and morphological entities in the White Sea. It remains to be documented whether the observed morphological and ecological differences are of a local character, or whether they have simply been overlooked in other contact zones.

## Introduction

Cryptic or sibling species are species that are difficult or impossible to distinguish based on morphological characters [1]. The existence of such taxa may reflect either inadequate exploration of the morphology, or differences in habitats, life histories or chemical recognition systems that have evolved without parallel divergence in morphology [2]. Often, with time and further research, diagnostic morphological characters will also be revealed, and the species would then be characterized as pseudo-sibling species [2]. Molecular characters have now revolutionized low-level taxonomy and disclosed a host of “molecular” cryptic species diagnosed by these characters alone [3]. Likewise one can expect that the description of such taxa will be followed by subsequent discovery of unique phenotypic traits, both morphological and biological.

The Pacific mussel *Mytilus trossulus* Gould (**MT**) was one of the first examples of marine cryptic taxa revealed by molecular genetic methods. Previously indistinguishable from the common mussel *M*. *edulis* L. (**ME**), MT was discovered in the 1980s by allozyme analysis [4–6]. A bulk of comparative biological and molecular data on these two taxa has been accumulated since then (see [7–9]).

According to the current zoogeographical view, MT and ME are relatively old lineages, which have diverged in allopatry in the Pacific and Atlantic oceans, respectively, since the Pliocene [9]. Subsequently, Late Pleistocene or Holocene trans-Arctic migration(s) have brought MT into the Atlantic [10–12]. Nowadays MT is widespread in the North Atlantic from the Gulf of Maine to the Arctic along the North American coast, and within the Baltic Sea and several isolated locations along the coasts of NE Europe [5,13–15]. In most cases wherever MT is found in the Atlantic, it occurs together with ME. In spite of the substantial molecular divergence in allozyme characters and in mitochondrial and nuclear sequences[5,16–18], the external morphology of ME and MT is considered to be essentially similar with no known taxonomical diagnostic characters, even though specimens from allopatric populations have been probabilistically discriminated in multivariate morphometric analyses of shell characters [13,19]. Basic physiological differences in temperature tolerance have also been demonstrated, with ME thriving better in warm temperatures. These differences probably underlie the different macrogeographic distributions in NE North America, where the distribution of ME extends more to the south [20,21]. With respect to salinity, the MT population that (alone) occupies the inner Baltic Sea is locally adapted to the strongly diluted brackish environment [9]. On the other hand, no consistent physiological differences in respect to salinity have been found between ME and MT from North American populations ([22], but see [23]).

As a rule, where the distributions of ME and MT overlap, the species do hybridize and introgression takes place (i.e. further backcrossing does occur) [7,9]. The extent of hybridization in the contact areas varies regionally. The extreme examples are the hybrid zone at the entrance to the Baltic Sea, where most individuals are hybrids, on the one hand, and the NE American contacts where most individuals represent the pure parental species, on the other [9,15,24]. In general, if hybridization is restricted and does not break up the genetic integrity of species, some incomplete reproductive barriers between the taxa should exist. Poor gamete compatibility between species and poor survival of hybrid larvae have been suggested as the principal barriers to gene flow between ME and MT [25,26]. The (micro)spatial segregation of mussel species could be another powerful factor limiting interbreeding [27,28], and from the ecological competition theory one could expect some ecological divergence to evolve between sympatric lineages [29,30]. There is however so far very little information on this topic for the sympatric MT and ME occurrences. Nevertheless, in the North American zone of overlap, MT tends to dominate more exposed localities and ME the more sheltered ones [31].

The systematic and taxonomic status of ME and MT (and of the third sister species *M*. *galloprovincialis* Lamarck, which we do not consider here) have puzzled marine biologists since the very discovery of their molecular differences. In the 1990s, ME and MT were suggested to represent either separate species, semispecies, subspecies, or genetic or even ecological races (e.g. [8,32,33]). The debate has centered on the morphological similarity (lack of distinction) and proneness to hybridization (lack of reproductive isolation) between the taxa. Therefore the cryptic mussel species were listed by Knowlton [2] among the “most vexing taxonomic controversies in the Sea”. This debate seems to have calmed down later on and the current practice has largely converged to using binomial names of the taxa, as with full species.

However, there is evidently a persisting hesitation among marine biologists, especially in Europe, concerning the reality of MT and ME as separate species. This is best illustrated by the Baltic mussel, already identified as MT 25 years ago, simultaneously with the initial species description [5,6,13,33]. A large part of the papers on the ecology of Baltic mussels have continued to refer to them as *M*. *edulis*, almost as often as *M*. *trossulus* (see S1 Fig), which appears confusing particularly as the presence of MT and usage of its scientific name were relatively soon adopted in North Pacific and NW Atlantic studies.

**Fig 1:**
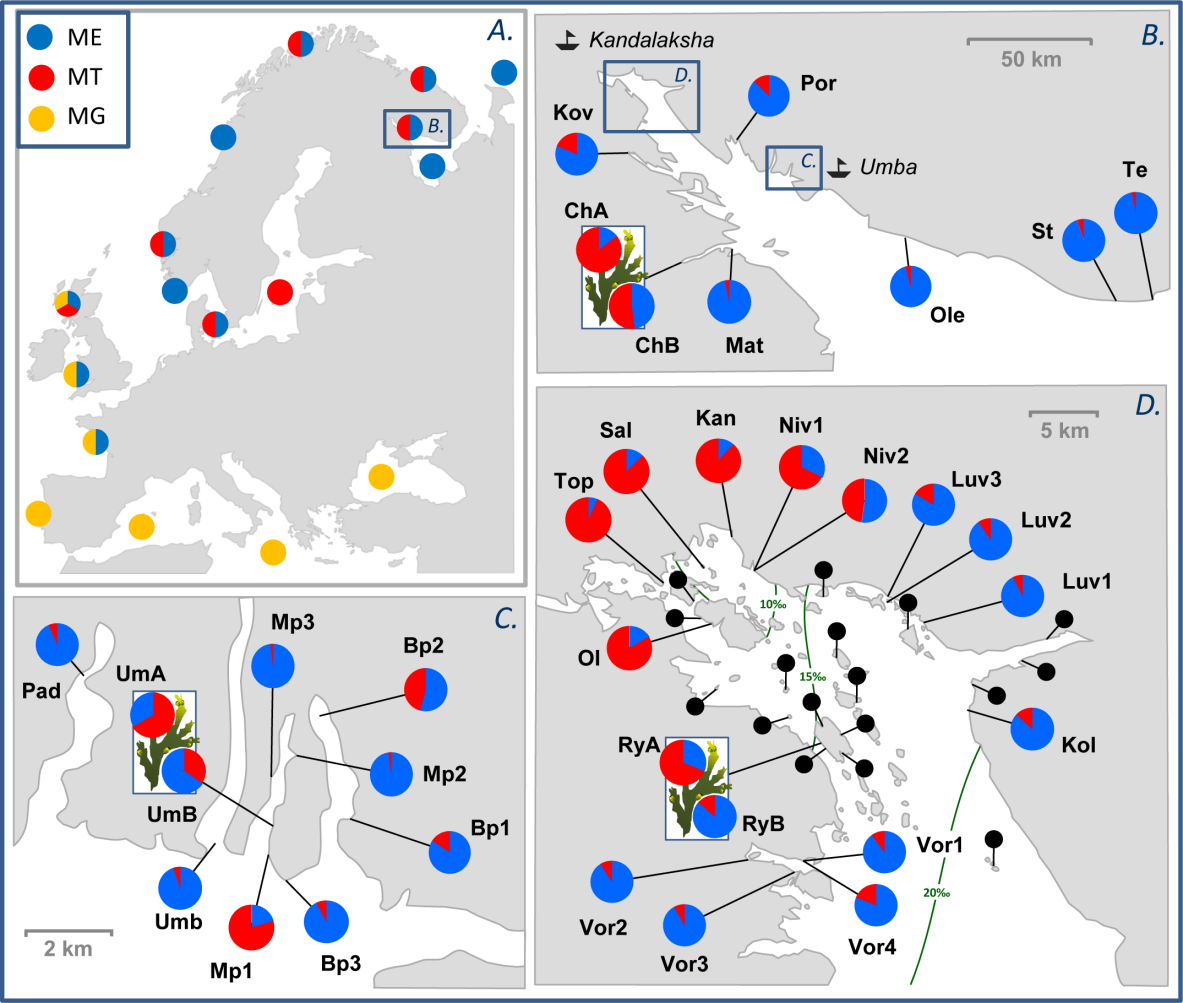
Map of study area and sampling sites. (A). Location of the Kandalaksha Bay of the White Sea, and general distribution of three mussel taxa in Europe: ME (*Mytilus edulis*, blue), MT (*M*. *trossulus*, red) and *M*. *galloprovincialis* (MG, yellow). Bi- or tri-colored circles indicate zones of sympatry (see references in the text). (B-D) Sampling locations in the White Sea: (B) Kandalaksha Bay. (C) Umba town area. (D) Top of Kandalaksha Bay. Pie diagrams depict estimates of the proportions of ME (blue sector) and MT (red sector) genomes in samples from the genetic dataset (GDS), obtained by STRUCTURE analysis of four-locus genotype data (PSS, see text for details). Data on paired local subsamples collected from the algal and the bottom substrates are shown above and below the algae pictogram, respectively. Black pins indicate sampling sites of the MDS (morphology only). Detailed sampling locality data are in S1 Table. The green lines in part D are isohalines of surface water (after [34]).

Apart from the Baltic Sea, occurrences of European MT have more recently been discovered in a number of isolated locations in Scotland, along the Norwegian coast, and in the Barents and White Seas in NW Russia [14,15] (see Fig 1A). Since the sampling so far has been rather limited, it seems plausible that the species would be even more widespread in NE Europe. Hence local marine biology in Scotland, Norway and NW Russia now faces the problems of identifying MT and ME in routine studies of northern *Mytilus* and of interpreting the results, particularly when presented in comparative framework with other regions. Are they just virtual entities lacking distinct morphology and ecology and interesting from a genetic or biogeographic point of view only, or true biological entities (species) that should be taken into account in any mussel oriented studies?

Aiming to clarify these questions we had in mind the simple concept that old evolutionary lineages such as ME and MT should ultimately demonstrate many characteristics of species, such as reproductive isolation in sympatry, ecological divergence and morphological diagnosability (secondary species criteria according to de Queiroz [35]). We performed a survey of populations from the White Sea Kandalaksha Bay where the presence of both species was disclosed in a previous study comprising only a few samples [15]. The sampling was now extended considerably and habitat and size differences were particularly targeted. Using data on four nearly diagnostic allozyme loci from the set initially used for the taxon delineation, and conventional statistical approaches applicable to any multilocus data, we assessed the extent of hybridization and estimated the proportions of purebred and hybrid individuals in mixed populations.

Further, we addressed two hypotheses on specific distinguishing features of the taxa. The first hypothesis was that ME and MT could be distinguished by a single morphological character: a distinct uninterrupted strip of a dark prismatic shell layer under the ligament on the inner side of the shell (“dark strip” further on). This character has long been used for discriminating MT from *M*. *galloprovincialis* in the Russian Pacific [36]; more lately it was suggested for identification of ME and MT too [37,38]. The second hypothesis was that sympatric mussels are segregated by the substrate to which they are attached by byssus threads: algal thalli (the fucoid brown algae *Ascophyllum nodosum* and *Fucus* spp.) vs. bottom substrates (stones, gravel, etc.). As far as we know, this hypothesis has never been tested before.

## Materials and Methods

### Ethics statement

Part of the collections of *Mytilus spp*. were performed within the Kandalaksha State Nature Reserve with an approval of the Reserve administration (agreements on cooperation between the Kandalaksha State Nature Reserve and the Department of Ichthyology and Hydrobiology, St. Petersburg State University Nos. 2010_30; 2011_24, 2011_25). No special permits were required for the field studies carried out outside the Reserve.

### Study area

**Fig 2:**
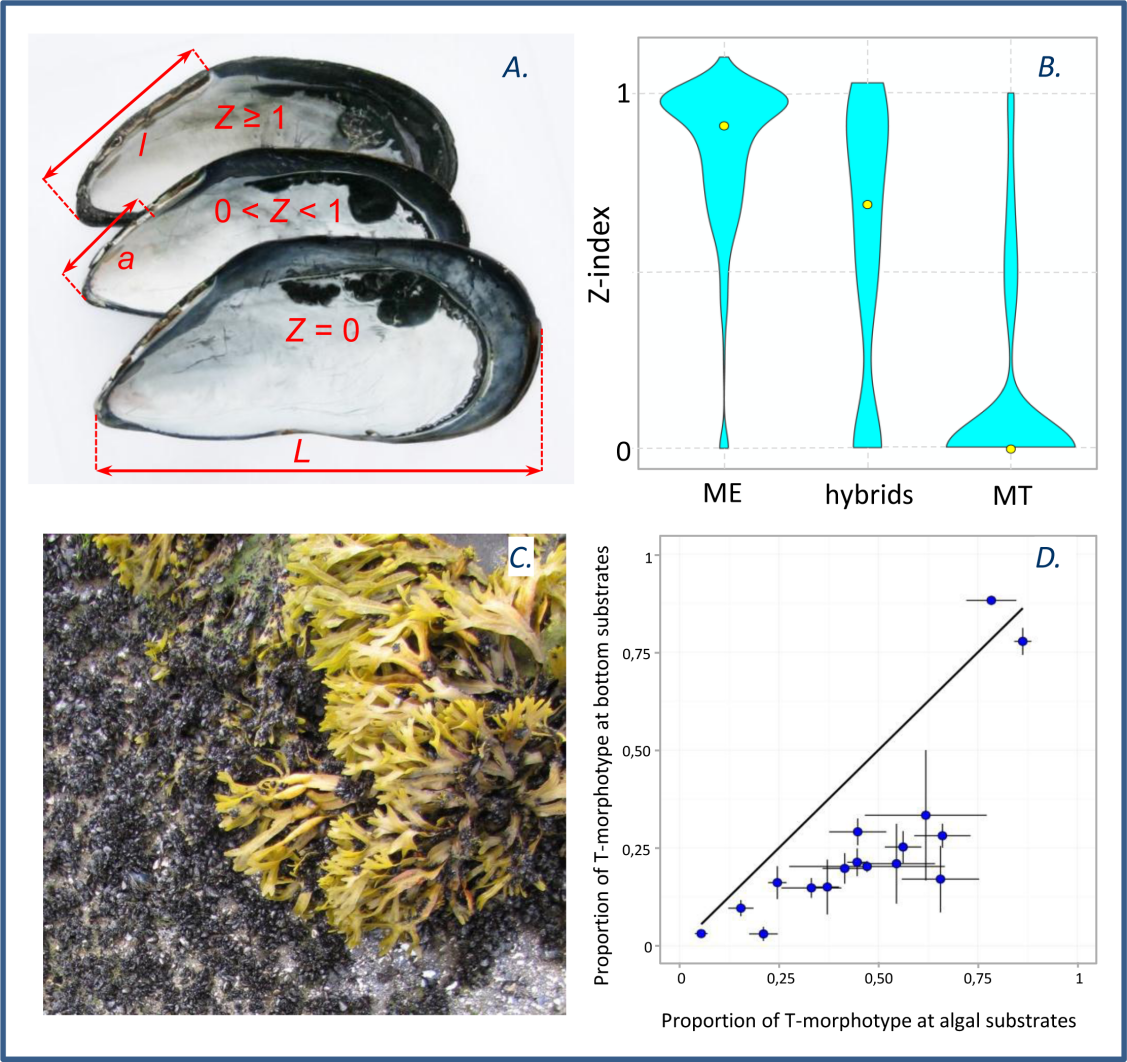
Mussel morphotypes and their distribution between different genotypes, and between algal and bottom substrates. (A). Mussel shells of different morphotypes: T-morphotype with an unbroken prismatic dark strip under ligament (lower shell) and E-morphotypes with the dark strip broken (middle) or absent (upper shell). Measurements indicated: L–total shell length, l–distance from shell umbo to the posterior end of the ligament, a–distance from umbo to the anterior end of the dark strip. The index Z = a/l; values of Z corresponding to the different morphotypes are shown. (B). The kernel density function of Z-values within the three genotypic classes (all samples pooled, the genotypic classes defined on the basis of STRUCTURE analysis, see text for details). Yellow dots indicate the medians. (C). Mussels growing on different substrates: on bottom ground vs. fucoid thalli. (D). The mean frequencies of T-morphotype (Z = 0) ± standard error on the algae (horizontal axis) plotted against that on the bottom in samples from 17 sites of MDS. If frequencies were identical on both substrates, the dots would fall on the diagonal (black line).

The Kandalaksha Bay of the White Sea (Fig 1) is a subarctic area characterized by a continental climate with cold winters (up to 5 months of ice cover) and warm summers. Mean annual sea surface temperature (SST) is 4.5°C and mean August SST is 13.8°C. In summer the salinity of surface waters is 24‰ in most of the Bay, but lower at the top influenced by freshwater outflow ([34,39], Fig 1D). There are few harbors in the area, the largest of them is Kandalaksha at the top of the Bay. Intertidal *Mytilus* mussels are found *en masse* both on brown algae and on the various bottom substrates (see Fig 2C), while subtidal mussel beds, each of which may cover dozens of hectares and feature biomasses of dozens of kilograms per m^2^, are mainly found in the top of the Bay and along its northern shore [40].

### Sample sets

Two sets of samples were used in our work. The main genetic data set (GDS) was used for the evaluation of genetic identities of individuals and populations, in order to analyze the extent of hybridization and of physical mixing of the taxonomic lineages within samples, and to reveal substrate preferences of genotypes and associations between genotype and morphology. An additional morphological data set (MDS) was used for the analysis of associations between mussel morphology and the substrate.

GDS included 31 geographical samples (*N* = 20–109, 1504 mussels in total). The material was collected in 2002–2013. Five of the samples were also analyzed earlier by Väinölä and Strelkov [15]. Most of the samples were taken at the intertidal, and a few samples from subtidal mussel beds (detailed sample information in Fig 1 and S1 Table). Each sample was collected from an area of approximately 5 m^2^, in such a way as to include mussels of different shell length (from 5 mm up) both from algal and bottom substrates (i.e. thalli of the fucoid brown algae *Ascophyllum nodosum*, *Fucus* spp. vs. stones, gravel, etc.). For three samples from localities geographically remote from each other–Umba (Um), Ryazhkov (Ry) and Chupa (Ch) (Fig 1)–the sampling procedure was modified to study the substrate specificity of genotypes: in these samples mussels from algal substrates and those from bottom substrates were kept as separate subsamples.

MDS included samples from 17 sites located all over the top of the Bay (Fig 1D, S2 Table) collected in 2011–2012. Sampling at each site was made within an area of approximately 10 m^2^ and consisted of six independent quantitative samples of mussels with a shell length ≥10 mm (*N* = 2–785; on average 139 individuals per sample, 14219 mussels in total). Three of six samples at each site were taken from the bottom substrates and three from algal ones. Samples from the bottom were obtained using a core frame of 16x16 cm, placed on a randomly selected mussel patch. To assess the abundance of mussels on the algal substrates we placed a frame of 50x50 cm on the fucoids, took out all the algae and then separated a small tuft selected at random. The tuft was weighted and so were the remaining algae from the core. The mussels from the tuft were counted and measured. The total mussel abundance in the frame area was estimated by multiplying the number of mussels from a selected tuft by the ratio of the total weight of algae in the frame to the weight of the selected tuft alone.

### Sample processing

#### Genotyping

Tissue samples for allozyme electrophoresis were taken from mussels that had either been kept alive or stored at -80°C. All GDS mussels were scored at a set of four nearly diagnostic allozyme loci, which exhibit 70–95% allele frequency differences between ME and MT: *Est-D*, *Gpi*, *Pgm*, *Odh* [15]. These loci were involved in the initial identification of the species differentiation and diagnosis between MT and ME [5,6,33]. The previous study of the White Sea mussels demonstrated good congruence between these allozymes and the PCR-based markers most commonly used for identifying ME and MT [15]. The assay conditions and nomenclature of alleles were as in [15].

#### Morphological measurements

Morphological analysis was performed for all MDS mussels and for 1049 mussels from the GDS samples (shells from eight genotyped samples were unavailable (see S1 Table), and some shells from the other samples were crushed during the analysis). We focused on the character “dark strip under the ligament”. In some mussels the light nacreous (or pearl) layer on the inner shell surface extends to the shell margin along the ligament (upper shell on Fig 2A). In others the nacreous layer by the ligament is underdeveloped and the underlying dark prismatic layer extends along the ligament as a separate narrow zone to make a broken or unbroken strip (Fig 2A, middle and lower shells). In highly pigmented shells this strip is very dark, nearly black in color. The following shell characteristics were measured with calipers to the nearest 0.1mm on the right shell valve: shell length (L), the distance from shell umbo to the posterior end of the ligament (l) and the distance from the umbo to the anterior end of a dark strip under the ligament (a) (a = 0 in case of an uninterrupted strip) (Fig 2A). The Z-index, Z = a/l, was defined to express the relative length of the dark strip under the ligament.

### Population genetic analysis

Two mutually complementary approaches to the analysis of population genetic data were used. First, the genotypic structure of individual populations was assessed using simple frequency estimates and conventional allelic and genotypic correlation statistics (disequilibrium), with reference to equilibrium expectations, and using a hybrid index approach based on direct allele counts. For this, a compound bi-allelic data set was employed (cf. [9,15,41]). Second, for the estimation of the genomic ancestries of individuals and classification of mussels into ‘purebred’ and ‘hybrid’ genotypic classes we also used the model-based Bayesian clustering method implemented in the program STRUCTURE [42]. The method infers the population structure based on multilocus genotype data on individuals, deriving their recent ancestry to a number of putative equilibrium parental populations each characterized by a set of allele frequencies at each locus (i.e. using multiallele data). Individuals in the empirical data set are probabilistically assigned to one of the parental populations or, in the case of admixed ancestry, jointly to parental populations.

#### Analysis of bi-allelic data

To obtain an informative bi-allelic data set, alleles at individual loci were classified and pooled into compound MT and ME specific alleles (hereinafter T- and E-alleles, correspondingly). Previously published data on pure MT populations from New Brunswick, Canada, and pure ME from Varangerfjorden, NE Norway (populations Nos. 3 and 6 in [15]) were used as reference. Each individual was characterized by the “T-score”–the number of its T-alleles summed across the four genotyped loci (ranged from 0 to 8). Every sample was characterized by the “T-frequency”: T-allele frequencies averaged over loci, scaled to the interval from 0 to 1 between reference frequencies from pure parental species.

The conventional population-level genotypic disequilibrium measures *F*_IS_ for individual loci (“heterozygote deficit”, reflecting the relative lack of heterozygotes versus the expectation under Hardy-Weinberg equilibrium) and *R’* for pairs of loci (relative “linkage disequilibrium”, reflecting the excess of two-locus heterozygotes versus random expectations) were calculated for each population, and averaged over loci or pairs of loci, as in Nikula et al. [43]. In equilibrium populations these statistics would equal zero while in mixed samples with an excess of parental genotypes they have positive values, the maximum possible value being 1 in the case of fully diagnostic loci. Since the loci studied were not fully diagnostic, we calculated in each case the maximum values of *F*_IS_ and *R’* as expected in hypothetical mixtures of the non-interbreeding ‘‘pure” taxa, which would have the same average allele frequencies (see [43] for details).

The extent of hybridization in populations was further illustrated by the distributions of individual T-scores. The observed distributions of T-scores in individual samples were compared with the corresponding predicted distributions in panmictic populations with the same average genetic composition on the one hand and in non-interbreeding mixed populations on the other hand. Predicted distributions were calculated as in [15]. The chi-square statistic was used as a measure of dissimilarity between observed and expected distributions and the Monte Carlo simulations (2000 replications) of Fisher's exact test were used for statistical testing.

In the above analyses the three pairs of subsamples collected from different substrates were analyzed both separately and pooled within the pairs to evaluate the influence of possible population substructuring on the hybrid score distributions, *F*_IS_ and *R’*.

#### Bayesian STRUCTURE analysis

For Bayesian assignment of the genotypic ancestries, the total data set (multiallele genotype data, no reference populations) was analyzed in STRUCTURE under the two-population admixture model using default parameters, with no prior information of the parental population identity; uncorrelated allele frequencies were assumed. 50000 MCMC replicates were conducted after discarding the first 30000 replicates as burn-in. Program was run multiple times to assess the repeatability of the results (results were always identical). The output was: (i) estimated allele frequencies in hypothetical parental populations (i.e. “pure” MT and ME), (ii) genomic ancestries of individuals–the estimated proportion of individual’s genotype inherited from parental populations (q-values, here called individual STRUCTURE scores (ISS) and attributed to the genome fraction in a range from 0 to 1 inherited from MT), and (iii) the estimated proportion of the MT genome at the population (sample) level (here called population STRUCTURE scores (PSS), and obtained by averaging ISS over a sample).

#### Classifying genotypes into purebred and hybrid classes

The inference of the hybrid vs. purebred ancestry of individuals from a limited set of incompletely diagnostic loci in a system where backcrossing may be extensive is necessarily subject to some uncertainty. Two pragmatic approaches were used to classify individuals to genetic classes of different ancestry. The first approach was based on T-scores. Considering the partially diagnostic nature of characters, we assigned mussels with T-score of 0 or 1 to ME, those with T-score of 7 or 8 to MT, and those with the intermediate T-scores (from 2 to 6) to hybrids. The performance of this procedure was assessed using sets of simulated genotypes of “known” ancestry (see the details below), following the approach of Vähä & Primmer [44] in terms of three statistics: efficiency–the proportion of individuals correctly assigned to a given ancestry class out of those actually belonging to this class; accuracy–the proportion of individuals correctly assigned to an ancestry class out of the total assigned to this class (correctly or incorrectly); and overall performance–the mean efficiency (averaged over the three classes) multiplied by the mean accuracy.

Another approach was based on the distribution of Bayesian ISS estimates in simulated samples of known ancestry (see S2 Fig and S3 Fig for details of the procedure and results). In short, we simulated samples from populations of a mixed ancestry involving six genotypic classes (MT, ME, their first and second generation hybrids, and first generation backcrosses to each species), composed so as to approximate their genotypic structure to the structure of empirical samples with approximately equal ME and MT ancestries in terms of the T-scores distributions and F_IS_ and R’ estimates (see Results section and S2 Fig for details). These simulated data were then analyzed along with the empirical data in STRUCTURE runs, to obtain distributions of ISS scores for “known” purebred and hybrid genotype classes in the context of the true empirical genotypic data. Threshold ISS values to effectively delineate the purebred vs. hybrid classes were then derived from these distributions by four alternative criteria based on the concepts of efficiency and performance (see above; S3 Fig): the thresholds between ancestry classes were adjusted so as to achieve 95% or 90% efficiency of identification of purebreds, or 95% or 90% efficiency of identification of compound hybrids. Of these alternative threshold criteria, the one that provided the maximal overall performance was then chosen and used for the assignment of the empirical mussels into putative ME, MT and compound hybrid ancestry classes by their ISS. We did not try to classify the hybrid genotypes in more detail since such a classification requires more loci [45].

### Associations between individual genotype, shell size, morphotype and substrate

We generally used a random-intercept, linear, mixed-effect model(LMM) [46] to assess associations between the measured genetic, morphological and ecological parameters. Samples in the case of GDS or sites in the case of MDS were considered as random factors in the analyses. The R programming language [47] with *nlme*-package [48] was used. Residuals plots were visually inspected to check for any deviations from homoscedasticity and normality of residual distributions. Wald Chi-square tests (Type II) implemented in the *Anova*-function in *car*-package [49] were used to assess the statistical significance of the model parameters. In those cases where ISS were used as dependent variables they were logit-transformed. In other cases where proportional data (percentages) were used they were φ-transformed.

#### Genotype vs. size

The association between ISS and shell length (L) in each sample of GDS was evaluated both with Pearson’s product-moment correlation (r) and, further, with LMM (ISS as dependent variable and L as independent fixed predictor; hereinafter Model 1).

#### Genotype vs. size and substrate

Three samples (Um, Ry, Ch), each consisting of two subsamples collected from different substrates, were used in the analysis. ISS was the dependent variable in LMM analysis and substrate type (Algae vs. Bottom), shell length L and their interaction were the independent predictors (Model 2).

#### Genotype vs. morphological character

Associations between ISS and the Z-index (reflecting the dark shell strip), and between L and Z-index in samples of GDS were analyzed using both Pearson’s r and LMM (Z as the dependent variable and ISS, L, and their interaction as independent predictors; Model 3).

From the above analyses the Z-index ranges characteristic of MT and of ME could be identified (see Results). Thereafter we classified all mussels into ME-like (E-) and MT-like (T-) morphotypes on the basis of their Z-scores. In a simple regression analysis, the frequencies of T-morphotypes in each sample were then regressed against the PSS of samples (Model 4).

The general performance of the classification of MT and ME by the morphological character was further tested in terms of efficiency, accuracy and overall performance, in analogy with the treatment of genetic ancestry classifications above.

#### Morphotype vs. substrate

The proportion of mussels with T-morphotype was calculated in each sample of MDS and used as the dependent variable in LMM with the substrate type as an independent predictor (Model 5).

We further tested whether the choice of substrate by one morphotype was independent of the abundance of mussels of the other morphotype. The log-transformed densities of T- and E-morphotypes (number of individuals per square meter) estimated from the MDS data were used for the analysis. The density of the T-morphotype was used as the dependent variable and the substrate type, density of E-morphotype and their interaction as the fixed part of the LMM (Model 6). The analysis was also performed using the density of E-morphotype as the dependent variable (Model 7).

## Results

### Genetic ancestry of populations

Sample- and locus-wise allele frequencies, frequencies of compound E-/T-alleles and PSS are presented in S1 Table. Estimates of *M*. *trossulus* background in terms of T-frequencies varied from 0.01 in Mp2 sample from the Umba area to 0.98 in the Top sample from the Kandalaksha area. The allelic composition of the two parental populations estimated by STRUCTURE was almost identical to that of the external reference MT and ME populations. The PSS values of the studied samples varied from 0.02 in Mp3 to 0.93 in Top and were very similar to the T-frequencies (S1 Table). Therefore we consider it sufficient to use the STRUCTURE score for describing the basic spatial pattern of variation.

**Fig 3:**
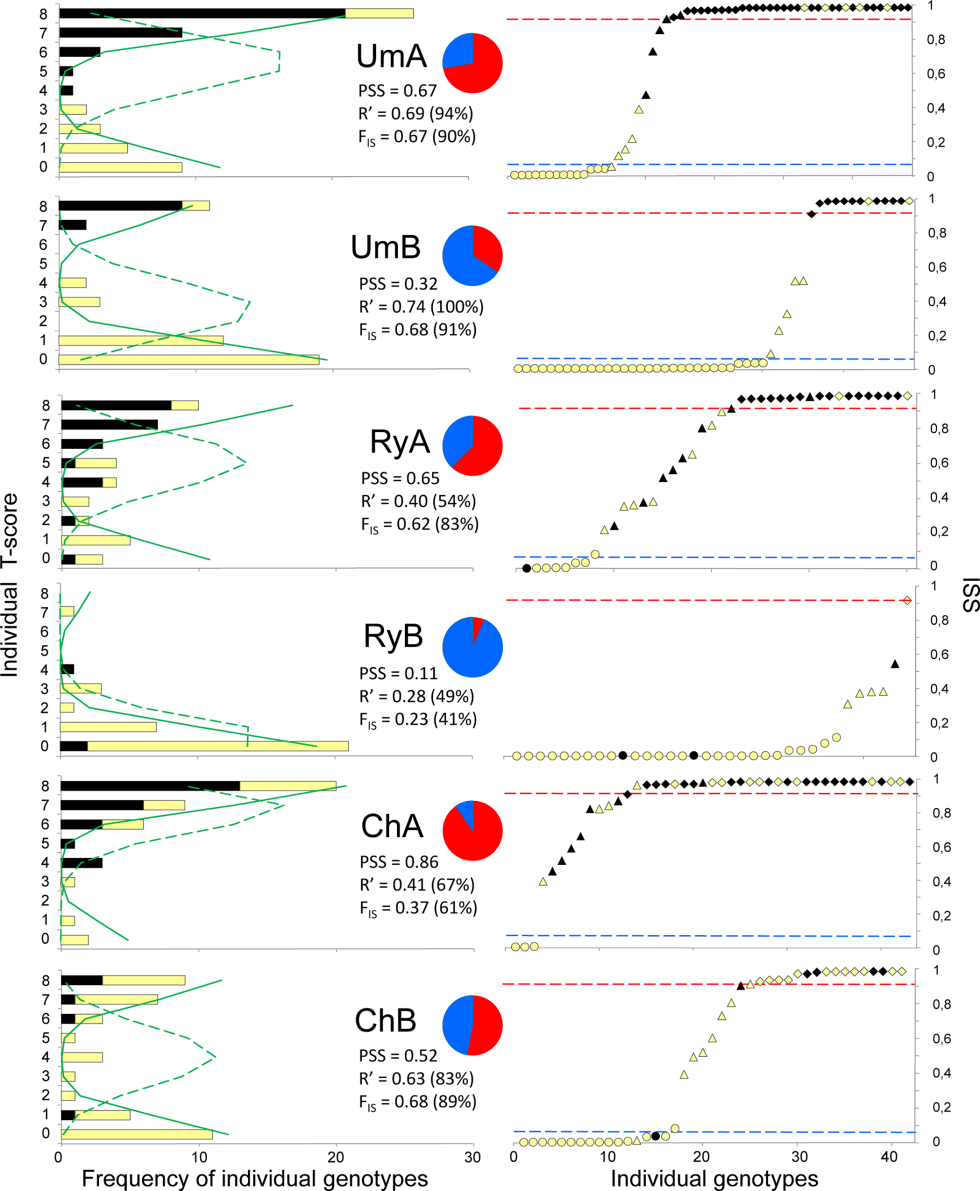
Genetic composition of mussel samples from Chupa (Ch), Umba (Um) and Ryazhkov (Ry). Subsamples collected from different substrates (A–algae, B–bottom) are treated separately. Left panel: Frequency distributions of individual T-scores (the sum of T-alleles across the 4 loci). Numbers of individuals are plotted on the abscissas, with T-scores as ordinates. Dark and light bars indicate T- and E-morphotypes, correspondingly. Green lines display the expected distributions under local random mating (dotted lines) and a mixture of parental genotypes without interbreeding (continuous lines). Central panel: Pie charts in the middle illustrate T-frequencies (red sector) vs. E-frequencies (blue sector). The estimated PSS values and the disequilibrium estimates R’ and F_IS_ are shown. Right panel: ISS distributions. Each symbol represents an individual, ranked along the horizontal axis by ISS. Dark symbols correspond to T-morphotypes, open symbols–to E-morphotypes. The shapes of the symbols reflect an individual T-score: circles– 0–1 (“ME”), triangular– 2–6 (“hybrid”), diamonds– 7–8 (“MT”). Horizontal lines reflect the thresholds chosen to delimit the genotype classes of ME (lower threshold) and MT (upper threshold) on the basis of the analysis of simulated samples (see text, S2 Fig and S3 Fig for details).

Overall, populations with high contributions of ME alleles (low PSS) dominated in the material. High contributions of MT alleles (high PSS) however were found in populations from the very top of the Bay, everywhere within and around the Kandalaksha harbor. MT genes were also common locally in Umba and Chupa, also within or in the vicinity of harbors. In all three geographical populations where subsamples from different substrates were treated separately, mussels from the algal substrates were dominated by MT alleles and those from the bottom by ME alleles. The absolute allele frequency differences were in tens of percent (Fig 1, pie diagrams on Fig 3, S1 Table).

### Genotypic structure of populations

**Fig 4:**
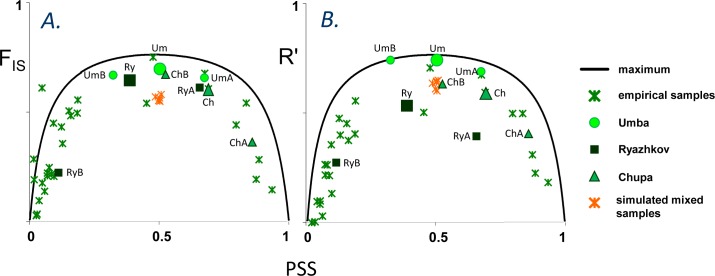
Distribution of genetic disequilibrium measures in samples of different genetic composition. PSS, characterizing samples’ genetic composition, are on abscissas, genotypic disequilibrium measures are on ordinate axes: (A). Intra-locus heterozygote deficit *F*_IS_. (B). Average inter-locus correlation *R*’. Green crosses correspond to empirical samples, orange crosses mark simulated mixed samples. Circles, squares and triangles mark Um, Ry and Ch samples, correspondingly (small symbols correspond to subsamples from different substrates, large symbols to pooled samples). The curves show the maximum disequilibrium in case of physical mixing without interbreeding. Expectations for equilibrium panmictic populations would be close to 0.

The estimates of genotypic disequilibrium *F*_IS_ and *R*’ (Fig 4, S1 Table) in samples with intermediate allele frequencies (PSS between 0.3–0.7) were always higher than 50% of the maximum level expected in a situation of a non-interbreeding mixture of parental taxa (on average 87% for *F*_IS_ and 83% for *R*’), which indicates some but limited hybridization. The plots of T-scores distributions also illustrated the limited hybridization. The distributions were bimodal, the genotypes with low and high scores dominating over the intermediates. In the diagrams of Fig 3 (left panel) the empirical distributions are compared with the predictions in panmictic and non-interbreeding mixture populations (see also S1 Table). Although the frequencies of intermediate T-scores were always much lower than expected in panmictic populations, they were nevertheless higher than expected in the mixtures of “pure” taxa with no interbreeding (S1 Table). In most comparisons the observed and predicted distributions were significantly different at *P* = 0.05 (Fisher’s exact test, S1 Table). The subsamples of Um, Ry and Ch from different substrates (A, B) showed no noticeable reduction of *F*_IS_ and *R*’ in comparison with pooled Um, Ry and Ch samples, indicating that microhabitat substructuring did not affect the ratios of hybrids in pooled samples much (Fig 4, S1 Table).

### Individual assignment

When classifying individuals by the direct allele count (T-scores), the percentage of putative ME (T-scores 0–1) varied among samples from 0% to 95% with a median of 70%, and that of putative MT (T-scores 7–8) varied from 0 to 78% with a median of 8%. The percentage of putative hybrids ranged from 5 to 38% with a median of 21% (S1 Table). Simulated genotypes were classified by this method with the overall performance of 76% (efficiency of 93%, 85%, 81% and accuracy of 96%, 97% and 72% for ME, MT and hybrids, correspondingly, see S3 Fig).

ISS estimates (Bayesian assignment of individual ancestry) showed good correlations with the T-scores within the samples (global Spearman’s *r* ≈ 1; see S1 Table). The intra-population ISS distributions were bimodal, dominated by scores close either to one or to zero; this is reflected in s-shaped cumulative distributions (right panel of Fig 3). Individuals classified as ME by their T-scores (0–1) had ISS less than 0.154, those classified as MT (7–8) had ISS higher than 0.909.

**Fig 5:**
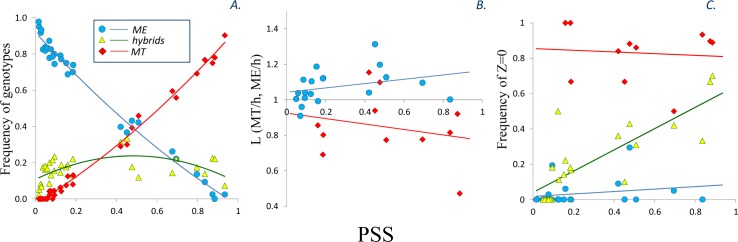
Taxonomic composition and morphological features of putative purebred and hybrid mussels in samples of different genetic composition. PSS on abscissas are plotted against: (A). frequencies of ME (blue symbols), MT (red symbols) and hybrids (green symbols) in samples; (B). ratios between sample means of shell length L of MT and hybrids (red symbols) and ME and hybrids (blue symbols) in samples; (C). frequencies of T-morphotypes among ME (blue symbols), MT (red symbols) and hybrid (green symbols) genetic classes in samples. Polynomial (A) or linear (B, C) functions were fitted to the data. Groups of less than 4 genotypes are not included. Classification of genotypes to ancestry classes in all cases was ISS-based.

In the exercise of classifying individuals into purebred and hybrid classes by their ISS, the simulated purebred ME had ISS range of 0.011–0.125 (median 0.012), purebred MT– 0.651–0.990 (median 0.987), simulated hybrids had ISS of 0.031–0.989 (median 0.521). Setting ISS thresholds for practical classification, the best overall performance (82%) was obtained when the criterion was to achieve 95% efficiency of identification of both MT and ME (efficiency of identification of hybrids in this case was 83%, accuracy of identification of ME, MT and hybrids—97%, 95% and 80%, correspondingly, see S3 Fig). The thresholds between ME and hybrids, and between hybrids and MT were thus set at ISS values of 0.062 and 0.916, respectively. With these limits, the percentage of ME in the empirical samples varied from 0% to 98% (median at 74%), that of MT from 0% to 90% (median at 8%) and that of hybrids from 2% to 38% (median at 18%) (S1 Table). These values were close to those from the T-score approach. ISS-based classification was further used for discrimination of the three genetic classes in all the analyses below where ME, MT and hybrids were compared. Fig 5A shows the estimates of the three ancestry classes in the empirical material. Frequencies of the two pure species are negatively correlated and vary widely among samples, while the proportion of hybrids is almost equal in all the samples and independent of the proportions of the two “parents”.

### Associations between individual genotype, shell size, shell morphotype and the substrate

#### Genotype vs. shell size

In most of samples the mean shell length of ME was on average 20% greater than that of MT, and the hybrids were intermediate in size (Fig 5B). Thus estimates of correlation between ISS and shell length (L) were usually negative (S1 Table; for pooled data *r* = -0.25, P<0.0001), as also reflected in the LMM model results (Table 1: Model 1).

**Table 1:** Results of regression model fitting.

Model	Dependent variable	Fixed predictors	Estimated parameters of regression model	Random factors	Wald statistic	P-level
1	ISS (logit)	L	-0.076	GDS sample N = 26	55.442	<0.001
		Intercept	0.218			
2	ISS (logit)	Substrate__bottom_	-4.11	GDS sample N = 6	11.031	0.029
		L	-0.152		39.206	<0.001
		L x Substrate__bottom_	0.044		1.051	0.306
		Intercept	5.766			
3	Z-index	L	0.005	GDS sample N = 26	21.60	<0.001
		ISS	-0.558		706.081	<0.001
		L x ISS	-0.0029		2.7368	0.098
		Intercept	0.693			
4	Proportion of T-morphotype	PSS	0.709	None	62.323	<0.001
		Intercept	-0.014			
5	Proportion of T-morphotype (arcsin)	Substrate__bottom_	-27.75	MDS site N = 17	53.485	<0.001
		Intercept	84.988			
6	Density of T-morphotypes (log)	Substrate__bottom_	-2.357	MDS site N = 17	22.754	<0.001
		Abundance of E-morphotype	0.572		19.102	<0.001
		Substrate__bottom_ x abundance of E-morphotype	0.175		0.914	0.339
		Intercept	2.746			
7	Density of E-morphotypes (log)	Substrate__bottom_	1.856	MDS site N = 17	38.555	<0.001
		Abundance of E-morphotype	0.374		19.004	<0.001
		Substrate__bottom_ x abundance of E-morphotype	-0.166		3.462	0.063
		Intercept	4.405			

#### Genotype vs. substrate

In all pairs of sympatric subsamples from alternative substrates, mussels from algae (UmA, RyA, ChA) were dominated by MT alleles, but those from bottom (UmB, RyB, ChB) by ME alleles. There was a significant association between ISS and the substrate type (P = 0.029), with lower predicted ISS at bottom substrates than on algae (P<0.001). The interaction between shell size and the substrate in predicting the genotype was not significant (P = 0.306) (LMM Model 2, Table 1).

#### Genotype vs. morphotype

The Z-index showed a significant negative correlation with ISS in most samples (S1 Table; for pooled data r = -0.77 P<0.001), and a positive correlation with shell length L (S1 Table; for pooled data r = 0.28, P<0.001). Accordingly, LMM predicts a strong negative dependence of Z on ISS and a weak but significant positive on L (Model 3, Table 1).

Violin plots in Fig 2B show kernel density functions of Z-values within the three genotypic classes. Putative ME were characterized by Z close to one, putative hybrids demonstrated the whole range of Z, and putative MT most frequently had Z = 0. For further analysis mussels with Z = 0 (an unbroken “dark strip”) were attributed to a “T-morphotype” while those with Z > 0 (“dark strip” broken or absent), to an “E-morphotype”. A good correspondence between the morphotype and genotype is illustrated on the diagrams in Fig 3 and in Fig 5C, and by a strong correlation between the frequencies of T-morphotype and PSS (Model 4, Table 1). Mussels classified as MT had high percentages of T-morphotypes (50–100% in different populations, median 88%) and ME had low percentages (0–29%, median 0%). The percentages did not vary much among samples with different genetic constitution (Fig 5C). The situation with putative hybrids was different however. In predominantly MT samples (high PSS) the hybrids were morphologically MT-like (percentage of T-morphotype up to 75%) while in ME-dominated samples (low PSS) they were morphologically ME-like (low proportion of T-morphotype, down to 0%) (Fig 5C).

The efficiency of the MT–ME classification using the single morphological character was 80% and 97% for putative MT and putative ME, respectively, when calculated for the pooled data. Thus 80% of the mussels genetically classified as MT have T-morphotype, while 97% of the mussels genetically classified as ME have E-morphotype. The accuracy was 75% for MT and 74% for ME, i.e. 75% of mussels with an unbroken dark strip were genetically classified as MT and 74% of mussels without the strip were assigned by genetic data to ME. The overall performance of the method was 66%.

#### Morphotype vs. substrate

In the non-genotyped MDS data set, the proportion of T-morphotypes was significantly related to the type of substrate (P<0.001, LMM, Model 5, Table 1): T-morphotypes were more common on the algal substrates than on the bottom substrates (Fig 2C). The analysis of absolute abundances (Models 6 and 7, Table 1) corroborates this tendency: the density of T-morphotypes was lower on the bottom, whereas the density of E-morphotypes was lower on the algae. Importantly, neither of these models revealed any significant interaction between the substrate type and the abundance of mussels of the “alien” morphotype.

## Discussion

This study characterized the geographical distributions of ME and MT in the Kandalaksha Bay of the White Sea, documented a pattern of mutual microhabitat segregation of these cryptic taxa by the type of substrate and confirmed the presence of a pronounced difference between them in a single morphological character. We analyzed the structures of the mixed populations and the extent of hybridization using a set of codominant marker genes, to achieve estimates of the frequencies of purebred and hybrid individuals at these sites.

### Extent of hybridization

In mixed populations purebred individuals always constituted the majority and putative hybrids only the minority (2%–38% of individuals, median 18%, as classified by STRUCTURE approach). This kind of distribution has been referred to as a bimodal hybrid zone [50]. Due to the strong tendency to bimodality, the frequency of hybrids did not vary much while the frequencies of parental forms varied strongly.

The definition of “pure” populations is a complicated issue, which we addressed with various pragmatic probability-related approaches. Employing the simulation data, pure ME and MT from the 6 synthetic samples were characterized by the mean ISS of 0.019–0.021 and 0.970–0.979, correspondingly. The STRUCTURE procedure is inherently biased to estimate a non-zero proportion of mixture even from by-definition-pure samples [51]. Accounting for this, empirical populations with an estimated 2–3% contribution from the “alien” species genome can be safely considered as pure. This applies to four of our White Sea samples, which can thus be considered pure ME (Mp2, PSS = 0.019; Mp3, 0.017; St, 0.027; Ma, 0.030). No pure MT samples were however found (the maximum PSS were 0.874 in Sal, 0.885 in Kan and 0.935 in Top). Since very few ME genotypes were recorded in the listed MT-dominated samples (S1 Table), we hypothesize that all MT populations in the area are introgressed.

It is difficult to compare the extent of hybridization in the White Sea with data from contact zones of other regions since different markers and statistical methods were often employed. The data of Väinölä & Strelkov [15] from a number of Fennoscandian populations are however directly comparable (using the same loci and statistics), and demonstrate a geographical trend in genomic mixing. This is reflected in the *F*_IS_ and *R*’ disequilibrium estimates, which in mixed samples from the White and Barents Seas (Russia) always exceeded 50% of the maximum, were somewhat weaker (around 50%) more to the south in Norway, and only 10–20% at the entrance to the Baltic Sea. Correspondingly, the distributions of individual T-scores changed from strongly bimodal in the White and the Barents Seas to a flat one in the Baltic–North Sea transition. Our broader data corroborate the inferences as regards the White Sea populations. The mixed populations in Scotland and the Northwest Atlantic can be compared only in terms of hybrid scores analogous to our T-scores. These suggest that 13–26% of individuals in North America [52–55] and 44% in Scotland (Fig 1 in [16]) are putative hybrids, compared with 5–38% (median of 21%) in our White Sea data. To sum up, hybridization between ME and MT is limited in Northern Russia and NE North America, extensive at the Baltic entrance and intermediate in Norway and Scotland (although only a few samples from the latter two regions have been investigated).

The low frequency of hybrids suggests a presence of powerful but incomplete reproductive isolation barriers between ME and MT in the White Sea. Theoretically, both prezygotic and postzygotic barriers are expected to act in bimodal hybrid zones [50]. From studies of the contact zone in the Northwest Atlantic several types of barrier have been suggested which could potentially act in the White Sea too: (i) Hybrid unfitness. Larvae from experimental heterospecific crosses show increased rates of abnormalities and increased mortality relative to conspecific crosses [26,56]. (ii) Spawning asynchrony. There is partial non-overlap of ME and MT spawning periods, as spawning of MT lasts longer [57,58]. (iii) Gamete incompatibility. In a number of experiments some individuals demonstrated a very strong block to heterospecific fertilization, while others were partially or completely compatible [25,26,55,59]. (iv) Habitat specialization. Distributional data suggest that salinity (MT preferring more saline localities), wave exposure (more exposed sites for MT) and depth (shallower littoral habitats for MT and deep sublittoral for ME) may serve as possible factors of spatial segregation [31,53,54,60], although the information is sometimes contradictory [23,61].

Microhabitat segregation might also act as an isolation barrier in mussels. In marine broadcast spawners the sperm retain fertilization capacity only for a few minutes. Therefore, depending on hydrodynamics, even distances as short as decimeters could be critical for fertilization success [62,63]. However, there is little information on microhabitat segregation in mussels ([28], see also below).

### Segregation by the type of substrate

Our results show that in the White Sea two basic types of mussel substrate, the algal (fucoid thalli) and the bottom (mud, send or pebbles), are exploited differently: MT predominate on the algae and ME on the bottom. To our knowledge this substrate hypothesis of ecological segregation has never before been considered, even though habitat differences between ME and MT have been addressed in many studies. There are however data on differences in some other traits that seem consistent with such a microdistribution pattern. Firstly, MT are generally lighter and smaller, and have thinner and more fragile shells than ME [14,64–66]. We also noted differences between ME and MT in the mean shell length in the White Sea (Fig 5B). Since mussels with fragile shells appear to be more sensitive to the wave impact, the algal thalli could serve as a shock-absorber for them. On the other hand, larger mussels with more massive valves can be shaken off the algae or press the algae down to the bottom.

Secondly, ME and MT differ in their aggregation behavior: ME tend to live in clumps unlike MT [28]. Aggregation behavior in mussels is thought to represent a complex adaptation that promotes efficient occupation of solid surfaces, prevents dislodgement by water currents and reduces predation risk in particular from sea stars [67,68]. *Asterias rubens* sea stars are indeed among the main predators of mussels in the White Sea [69]. Hence ME with an aggregative strategy would benefit from establishing clumps on the bottom surfaces. On the other hand, for MT living on algae could reduce the risk of contact with a creeping predator. Lowen et al. [70] demonstrated experimentally that MT are more vulnerable than ME to sea star and crab predation, having weaker predator-induced defenses, such as weaker byssal attachment, a smaller adductor muscle and absence of shell thickening in the presence of predators. The algae that rise above the bottom level at high water may grant MT an asylum against the sea stars.

In all, considering the other biological differences documented between ME and MT, their partial substrate-related segregation would most likely seem to be caused by wave exposure and predator avoidance. Theoretically, selective larval settlement, post-larval active habitat choice and direct selection by predators and/or by wave exposure could be the actual mechanisms leading to the observed non-random mussel distribution. The relative importance of these mechanisms cannot be judged from our results alone.

An interesting question still to be answered is whether MT and ME exploit algal and bottom substrates differently in allopatry. From our preliminary observations, the choice of the substrate by mussels of the E- or T-morphotypes does not depend on the abundance of mussels of another morphotype. This can indicate that ME universally prefer the bottom substrates while MT–the algal ones.

### Regional distributional pattern

In the previous genetic survey of the White Sea mussels, with a broader overall geographic extent but sparser sampling [15], just a single MT-dominated population was identified, in Umba, and in other regions dominated by ME only scattered observations of MT or its genes were made. We now find that MT occupies the whole brackish top of the Kandalaksha Bay, and is also locally numerous in the Chupa and Umba areas, which are not brackish at all [34]. On the other hand, Kandalaksha, Chupa and Umba are all urban settlements and historically important harbors (although there is now little shipping at the latter two localities). The confinement of MT to the historical harbor areas seems to support our hypothesis that MT has been introduced into the White Sea–Barents Sea region and its further expansion within the region facilitated by ships [15], even though the initial source of introduction remains unknown. As with the White Sea, in the Barents Sea MT is also currently known mainly from harbor areas ([15]; Fig 1A).

The proposition that MT has successfully invaded harbors in a region inhabited by a congener may indicate that MT is a more opportunistic species than ME. Indeed, some differences in the life-history traits accord with this hypothesis. Apart from the differences in size and weight, in the North American contact zone MT produce a larger amount of smaller eggs than ME [57,71]. Therefore, MT would have a relatively high fecundity, a short generation time, and high intrinsic rates of population increase–all features that are characteristic of opportunistic species [72]. In this context the association of MT with the algal thalli could also be a manifestation of its opportunistic life-strategy since intertidal algae make a relatively ephemeral and unstable microhabitat, disturbed by storms, freezing and ice scrubbing.

Another intriguing question is whether the MT populations in Chupa, Kandalaksha and Umba are genetically isolated or connected by gene flow over the intervening ME populations. Since the estimated dispersal distance of *Mytilus* larvae is 20–50 km [73,74] and the straight-line distances among the three towns are about 70–100 km (see Fig 1), we surmise that these populations are only weakly connected if at all. Providing that other similar occurrences indeed do not exist in the region, they are likely to be mutually independent or semi-independent populations.

### The White Sea contact zone vs. other *Mytilus* contacts

The contact areas of MT and ME (or *M*. *galloprovincialis*) have mostly been considered as hybrid zones (e.g. [9,33,53,75,76]). A basic (tension) hybrid zone model assumes that the zone is maintained owing to a balance between the selection against hybrids vs. the continued dispersal of parental genotypes into the zone from the outside [77]. Most *Mytilus* contact zones are classified as mosaic hybrid zones [9,75,76,78,79]. In such zones the parental species inhabit separate habitat patches of a size larger than the scale of population dispersal, and hybridization occurs at the boundaries of the patches or in intermediate habitats [80]. Nevertheless, any hybrid zone model explicitly assumes that the system can exist only in a broader context of parapatry, requiring an influx of parental species from the surrounding pure populations.

The patchy distribution of the White Sea MT is obvious, but does the pattern fit the hybrid zone context? The area of co-occurrence near Kandalaksha would seem to be a hybrid zone between the parapatric MT populations in the top of the Bay and ME populations in its open parts (Fig 1D). However, in Umba we found a few patches of MT in the heart of an ME-dominated area (Fig 1C), so that the spatial pattern seems more like sympatry than parapatry, taking into consideration larval dispersal distance in mussels (see previous section). This pattern is probably not unique but also encountered in other *Mytilus* contacts, such as that between ME and *M*. *galloprovincialis* in France [27,78]. Nevertheless, regardless of whether the mixed ME–MT populations always function as hybrid zones or not, the situation could alternatively be described as one where two sympatric species capable of introgressive hybridization co-occur. In such a case the species could evolve niche segregation which would then further promote their reproductive and demographic independence; the White Sea ME and MT indeed demonstrate such segregation by the type of substrate.

Alternative to the hypothesis of post-contact evolution of niche segregation (e.g. as an outcome of competition and(or) selective reinforcement of prezygotic barriers to avoid investment to unfit hybrids) the ecological differences in MT–ME contact zones could represent specialization that already evolved during the isolation of the lineages when they diverged in allopatry, prior to the invasion of the Pacific MT to the Atlantic basin. Distinguishing these hypotheses is fundamental to the understanding of the nature of ecological differences that maintain the zones. In the latter case the habitat specialization of lineages should be similar in different contact zones, but in the former case not necessarily so.

Pronounced differences between different ME–MT contact zones are exemplified in a comparison between the two best studied zones, those in the Baltic Sea and in the northeastern North America. At the entrance to the Baltic Sea, ME and MT are geographically segregated by salinity along a strong environmental gradient, the area of ongoing hybridization is narrow, hybridization is extensive (a situation that fits the basic hybrid zone model well [33]) and so is introgression ([9,15] and references therein). (Yet to note, recent genomic data [17,18] suggest that the extent of nuclear introgression into the Baltic MT is substantially lower than was previously suspected on the basis of data on fewer DNA markers [9]). By contrast, in the Atlantic North America the two lineages are distributed in a mosaic fashion. Mixed and pure populations of both species alternate at regional and local scales, no clear segregation of lineages by salinity is observed, hybridization is limited and introgression is negligible ([9] and references therein). With respect to the extent of hybridization and the regional spatial organization, the White Sea contact zone seems to be more similar to the North American one ([15]; this study). It rather seems that the Baltic–North Sea cline is not typical as a marine contact zone, in contrast to the North American and White Sea zones, and probably to the Norwegian and Scottish ME-MT contacts too [14,15]. Nonetheless, without direct comparative data it is not yet safe to extrapolate our findings on ecological assortment of ME and MT in the White Sea to the American, Norwegian or Scottish populations, or vice versa.

### “Dark strip”, a nearly diagnostic character

Morphological differences between ME and MT have been addressed in many investigations [13,19,65,81]. Although some characters, e.g. hinge plate length, anterior adductor scar length, and shell proportions show some statistical differentiation, the differences between ME and MT in particular morphological characters are small and much less reliable than genetic markers [19]. Interestingly, shell shape differences were more pronounced in ME and MT growing under artificial conditions of suspended aquaculture [14,82]. Therefore morphological species identification has mostly been considered based only on multivariate techniques [13,83].

The “dark strip” was first mentioned as a typical character of Pacific MT in the 1980s [36], and has since then been used as a diagnostic quantitative [37] or qualitative [38] character to distinguish MT vs. *M*. *galloprovincialis* in the Russian Far East. However, its utility for discrimination of these species from other geographical regions and between these species and ME has remained hypothetical. This hypothesis was partly supported in a study of small (N = 10) samples of genotyped mussels from allopatric populations [84]. Yet no attempts to apply this trait to discrimination of mussels of known genotypic ancestry from zones of sympatry have been made.

Our data suggest a strong diagnostic capacity of the character: T-morphotype was found in 80% of MT and only in 3% of ME. As far as we know, there is no other single qualitative morphological character with such a strong discriminative power in any sympatric MT–ME populations. However, it remains unclear whether the “dark strip” can be used to discriminate between ME and MT in geographical regions other than the White Sea.

The adaptive significance of the character, if any, is also unknown. The absence of the white nacreous layer along the ligament exposing the underlying dark prismatic layer is possibly associated with a weak development of the nacre in the whole shell. Nacre plays an important role in ensuring strength and resilience of shells [85]. Its underdevelopment might be the reason of both the extension of prismatic layer along the ligament and the relative fragility of MT shells.

### Consistently separable biological entities

With the progress in molecular systematic techniques and the parallel advances in biodiversity theory, we have become aware that much of the true biotic diversity has been hidden at the level of (pseudo)sibling species, which are easier to recognize by molecular than by morphological characters (e.g. [2]). It has also become evident and generally accepted that many “good” species do undergo introgressive hybridization without broader harm to their taxonomic identity or integrity (e.g. [86]), and that the geographical distributions of many species are now influenced by humans rather than solely by climate, geography and other natural factors [87].

ME and MT in the White Sea are a good example of the veracity of these three tenets. They do hybridize, with hybrids constituting about 1/5 of individuals in mixed populations. On the other hand, this rate does not seem excessive since many contact zones between well-defined species are dominated by hybrids rather than parents (cf. [50]). ME and MT have rather similar shell morphologies and the best taxonomic character known to date, the “dark strip” [38], discriminates purebred ME and MT from mixed populations with an efficiency of 97% and 80% and an accuracy of 74%, 75% for ME and MT, correspondingly. Regional distribution of MT is probably governed not so much by natural ecological factors as by the marine traffic. Actually, MT in the White and Barents Seas stands out as a “harbor mussel”, which has presumably been distributed by ships ([15]; this study).

Being ecologically close species *a fortiori* capable of limited hybridization but still consistently retaining their identities, ME and MT should somehow divide resources and space in sympatry and possess some habitat post-zygotic isolation barriers. In sedentary organisms such as mussels, the substrate of attachment is the critical element of habitat. Indeed, ME and MT are segregated by the type of substrate, with MT being more common on algal and ME on bottom substrates. We may expect that the White Sea ME and MT will also show many other inconspicuous phenotypic and ecological differences both in natural habitats and in aquaculture, as indeed has been demonstrated for some characters in other mussel contact zones (see references above).

In conclusion, ME and MT are here shown to represent clearly distinguishable genetic, ecological and morphological entities in the White Sea, even if not fully discrete. This taxonomic dualism of the “common mussel” should henceforth be recognized in any mussel-oriented studies in the area of distributional overlap. So far not a single one out of the 13 papers on the ecology and physiology of the Kandalaksha Bay mussels published since 2012 even mentions MT (the presence of which was first internationally published in 2011 [15]; paper count based on a Scopus database search, cf. S1 Fig).

For decades it has remained intriguing that ME and MT are morphologically so close and yet so indeterminate in respect to the pattern and extent of habitat specialization in their contact zones, in spite of representing independent evolutionary lineages of considerable age [9]. Our study introduces an alternative question: why are they so consistently different in the White Sea? Were the basic morphological and habitat differences revealed in our study overlooked in other populations and contact zones, or have we described a locally restricted phenomenon? Until parallel morphological and ecological analyses in other contact zones are undertaken, it would be prudent to avoid simplistic extrapolation of the White Sea results to contact zones and other populations in other geographical regions.

## Supporting Information

S1 Fig Scientific names applied to the Baltic *Mytilus trossulus* in the non-genetic papers focused on mussels in recent years. OX–year of publication, OY–number of papers. Four categories of names are depicted: "blue mussel", "*Mytilus edulis*", "*Mytilus trossulus*" and "complex names" (*Mytilus sp*., *Mytilus spp*., *Mytilus edulis trossulus*, *Mytilus trossulus* x *M*. *edulis*). The graph is based on the results of a search in Scopus for papers with words “Baltic AND Mytilus OR mussel” in title, abstract or key words, published through 1998–2014; only non-genetic papers dealing with blue mussels from the Baltic Sea excluding Kattegat, the Straits and the Kiel Bay (areas dominated by *M*. *trossulus*, e.g. Väinölä & Strelkov 2011, Zbawicka et al. 2014). There is a significant negative trend in the frequency of the use of the name *M*. *edulis* as compared to other taxonomic (Latin) names with time (Spearman's r = -0.65, p = 0.006).
(TIF)

S2 Fig Genotype distributions of six simulated mixed samples: procedure and results. Mixed samples of known genotypic ancestry were constructed for a reference to test and refine methods of a-posteriori assignment of individuals to purebred and hybrid classes in the empirical data. The procedure was as follows: (1) Using the allele frequencies in parental populations as reconstructed by STRUCTURE, and a custom script to sample random multilocus genotypes, simulated samples were obtained separately from each of the six genotypic classes: MT, ME, their first and second generation hybrids, and first generation backcrosses to each species (similar to Nielsen et al. 2006, Molecular Ecology Notes 6(4):971–973). (2) Simulated samples of mixed ancestry and limited interbreeding (N = 200, six replicates) were constructed by mixing randomly chosen simulated individuals of the six genotype classes in proportions 80:80:10:10:10:10 (i.e. 40% each purebred, 20% various hybrids). These proportions were chosen to approximate the structure of simulated samples to the empirical samples with approximately equal ME and MT ancestries, as reflected in their T-score distributions and F_IS_, R’ estimates. (3) The simulated samples were analyzed in STRUCTURE runs together with the empirical data set. In each of six replicate runs, a different simulated sample was analyzed along with the empirical data (the set of simulated specimens added should be limited to exert the least influence on the classification of data in the analysis). From the analyses, distributions of estimated ancestries (individual STRUCTURE scores, ISS) for simulated individuals of known hybrid/non-hybrid ancestry were obtained, in the settings of the true data set. (4) From the simulated ISS distributions (data from the six runs pooled) thresholds for classifying the empirical individuals into purebreds vs. hybrids were derived using the procedure in S3 Fig. The figure displays the structure and statistics for each of the six simulated mixed samples, and the application of the classification criterion (see S3 Fig for details) to these data. Three diagrams are shown for each sample (cf. Fig 3 in the main paper): (1) The left panel shows the frequency distribution of the T-score, i.e. numbers of individuals with different number of T-alleles in their genotype. Colors represent the genetic ancestry classes in each column (see legend). (2) In the middle panel, the pie charts illustrate the composite T-frequencies (red) vs. E-frequencies (blue) in the samples. PSS, R’ and F_IS_ estimates are also indicated. (3) The right panel shows ISS distributions. Each symbol corresponds to an individual simulated genotype, ranked along the horizontal axis by ISS. Blue circles–ME, red diamonds–MT, yellow-green triangles–compound hybrids. Colored lines mark the operational thresholds for pure species/hybrid discrimination accepted according to the chosen criterion (see S3 Fig for the details).
(TIF)

S3 Fig Comparison of different methods of classifying individuals into purebreds and hybrids. The figure shows the estimates of: (A) efficiency; (B) accuracy and overall performance of different methods which assigned simulated genotypes of initially known ancestry to putative MT (red symbols), ME (blue symbols) and hybrids (green symbols). “Efficiency” is the proportion of individuals correctly assigned to a given ancestry class out of those actually belonging to this class; “accuracy”–the proportion of individuals correctly assigned to an ancestry class out of the total assigned to this class (correctly or incorrectly); and overall performance–the mean efficiency (averaged over the three classes) multiplied by the mean accuracy (cf. Vähä & Primmer 2006, Mol. Ecol.15(1):63–72). The five methods of classification (on abscissa) differed as follows: *Method 1*: Classification was based on T-scores. Individuals with T-scores of 0–1 were assigned to ME, those with T-scores of 7–8 to MT, and the rest to hybrids. Methods 2–5 used criteria based on the ISS (individual STRUCTURE scores). The threshold values of the scores were chosen so as to achieve a certain level of efficiency, either for the purebreds (MT, ME) or for hybrids (compound of 4 hybrid ancestry classes, see S2 Fig legend): *Method 2*–95% efficiency for assignment of purebreds. *Method 3*–95% efficiency for assignment of hybrids. *Method 4*–90% efficiency for assignment of purebreds. *Method 5*–90% efficiency for assignment of hybrids. The classification method (criterion) demonstrating the best overall performance (black solid line, method 2) was used for further assignment of empirical genotypes.
(TIF)

S1 Table Sampling localities and samples information for genetic data set (GDS). (XLSX)

S2 Table Sampling localities and samples information for morphological data set (MDS). (XLSX)
