# Supplementary figures and images for: Genetic, Ecological and Morphological Distinctness of the Blue Mussels Mytilus trossulus Gould and M. edulis L. in the White Sea
Source: PLoS One. 2016 Apr 4;11(4):e0152963. doi: 10.1371/journal.pone.0152963 (PMC4820271; doi:10.1371/journal.pone.0152963)

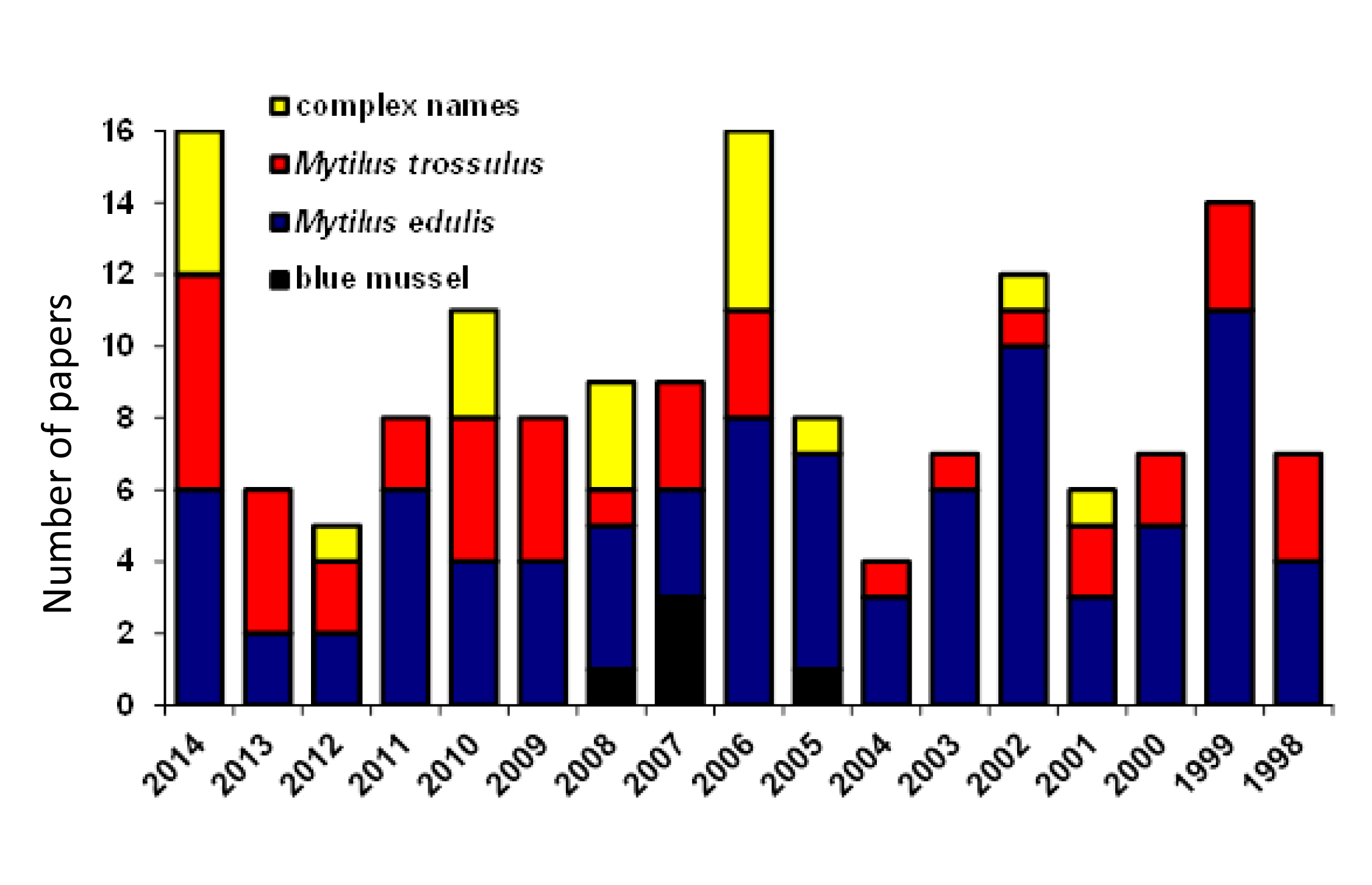

Supplement: S1 Fig — OX–year of publication, OY–number of papers. Four categories of names are depicted: "blue mussel", "Mytilus edulis", "Mytilus trossulus" and "complex names" (Mytilus sp., Mytilus spp., Mytilus edulis trossulus, Mytilus trossulus x M. edulis). The graph is based on the results of a search in Scopus for papers with words “Baltic AND Mytilus OR mussel” in title, abstract or key words, published through 1998–2014; only non-genetic papers dealing with blue mussels from the Baltic Sea excluding Kattegat, the Straits and the Kiel Bay (areas dominated by M. trossulus, e.g. Väinölä & Strelkov 2011, Zbawicka et al. 2014). There is a significant negative trend in the frequency of the use of the name M. edulis as compared to other taxonomic (Latin) names with time (Spearman's r = -0.65, p = 0.006). (TIF) [file pone.0152963.s001.tif]

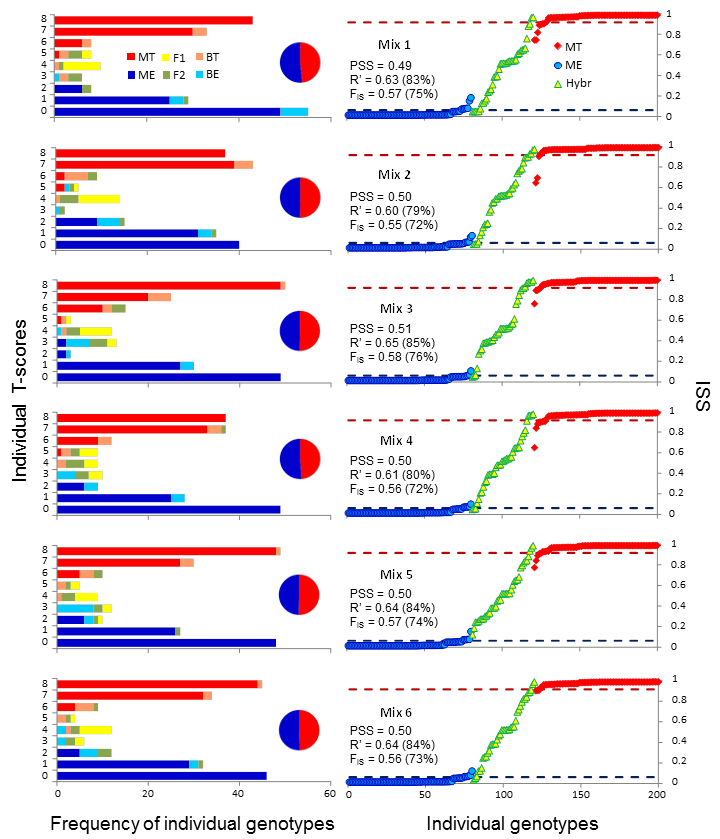

Supplement: S2 Fig — Mixed samples of known genotypic ancestry were constructed for a reference to test and refine methods of a-posteriori assignment of individuals to purebred and hybrid classes in the empirical data. The procedure was as follows: (1) Using the allele frequencies in parental populations as reconstructed by STRUCTURE, and a custom script to sample random multilocus genotypes, simulated samples were obtained separately from each of the six genotypic classes: MT, ME, their first and second generation hybrids, and first generation backcrosses to each species (similar to Nielsen et al. 2006, Molecular Ecology Notes 6(4):971–973). (2) Simulated samples of mixed ancestry and limited interbreeding (N = 200, six replicates) were constructed by mixing randomly chosen simulated individuals of the six genotype classes in proportions 80:80:10:10:10:10 (i.e. 40% each purebred, 20% various hybrids). These proportions were chosen to approximate the structure of simulated samples to the empirical samples with approximately equal ME and MT ancestries, as reflected in their T-score distributions and FIS, R’ estimates. (3) The simulated samples were analyzed in STRUCTURE runs together with the empirical data set. In each of six replicate runs, a different simulated sample was analyzed along with the empirical data (the set of simulated specimens added should be limited to exert the least influence on the classification of data in the analysis). From the analyses, distributions of estimated ancestries (individual STRUCTURE scores, ISS) for simulated individuals of known hybrid/non-hybrid ancestry were obtained, in the settings of the true data set. (4) From the simulated ISS distributions (data from the six runs pooled) thresholds for classifying the empirical individuals into purebreds vs. hybrids were derived using the procedure in S3 Fig. The figure displays the structure and statistics for each of the six simulated mixed samples, and the application of the classification criterion (see [file pone.0152963.s002.tif]

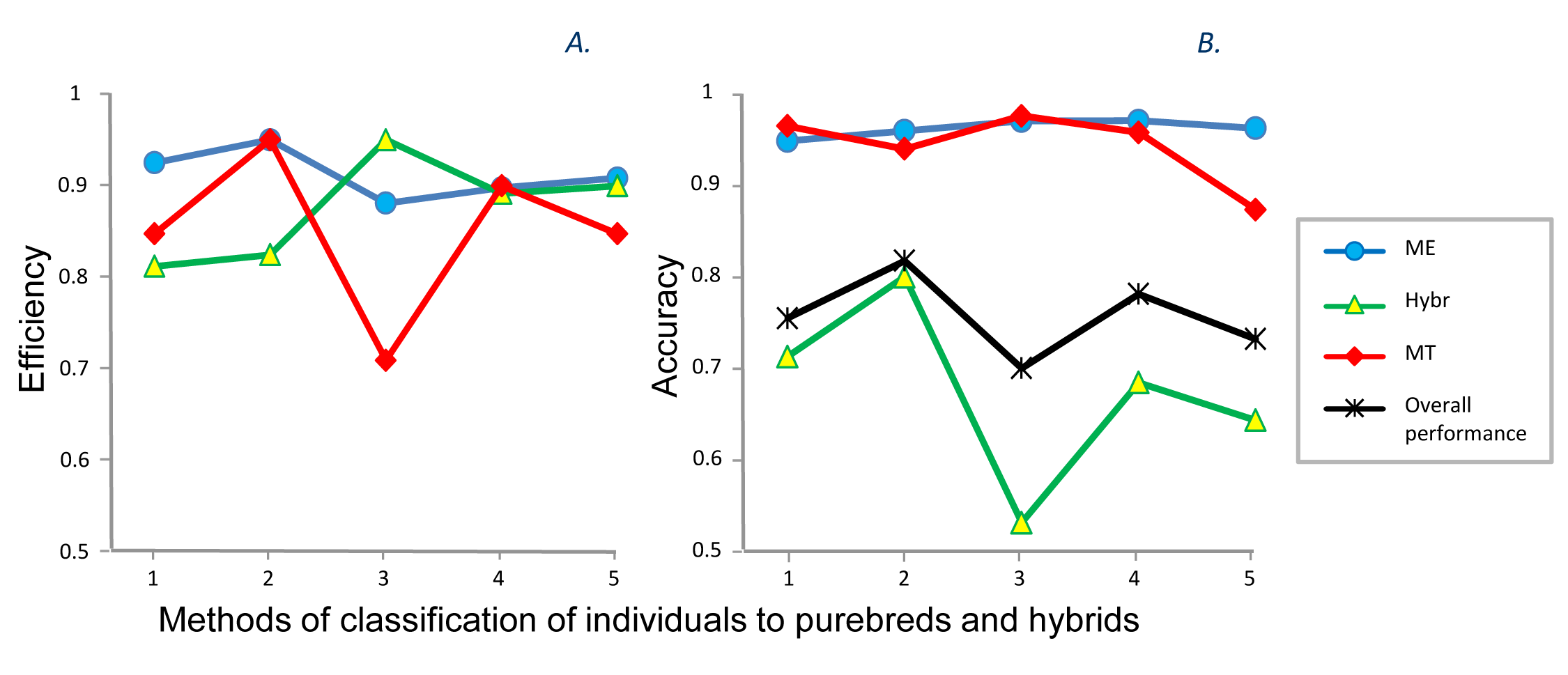

Supplement: S3 Fig — The figure shows the estimates of: (A) efficiency; (B) accuracy and overall performance of different methods which assigned simulated genotypes of initially known ancestry to putative MT (red symbols), ME (blue symbols) and hybrids (green symbols). “Efficiency” is the proportion of individuals correctly assigned to a given ancestry class out of those actually belonging to this class; “accuracy”–the proportion of individuals correctly assigned to an ancestry class out of the total assigned to this class (correctly or incorrectly); and overall performance–the mean efficiency (averaged over the three classes) multiplied by the mean accuracy (cf. Vähä & Primmer 2006, Mol. Ecol.15(1):63–72). The five methods of classification (on abscissa) differed as follows: Method 1: Classification was based on T-scores. Individuals with T-scores of 0–1 were assigned to ME, those with T-scores of 7–8 to MT, and the rest to hybrids. Methods 2–5 used criteria based on the ISS (individual STRUCTURE scores). The threshold values of the scores were chosen so as to achieve a certain level of efficiency, either for the purebreds (MT, ME) or for hybrids (compound of 4 hybrid ancestry classes, see S2 Fig legend): Method 2–95% efficiency for assignment of purebreds. Method 3–95% efficiency for assignment of hybrids. Method 4–90% efficiency for assignment of purebreds. Method 5–90% efficiency for assignment of hybrids. The classification method (criterion) demonstrating the best overall performance (black solid line, method 2) was used for further assignment of empirical genotypes. (TIF) [file pone.0152963.s003.tif]
